# Recombinant Phage Coated 1D Al_**2**_O_**3**_ Nanostructures for Controlling the Adhesion and Proliferation of Endothelial Cells

**DOI:** 10.1155/2015/909807

**Published:** 2015-05-19

**Authors:** Juseok Lee, Hojeong Jeon, Ayman Haidar, Hashim Abdul-Khaliq, Michael Veith, Cenk Aktas, Youngjun Kim

**Affiliations:** ^1^INM-Leibniz-Institute for New Materials, Campus D2 2, 66123 Saarbrücken, Germany; ^2^Center for Biomaterials, Biomedical Research Institute, Korea Institute of Science and Technology (KIST), Hwarangno 14-gil 5, Seongbuk-gu, Seoul 136-791, Republic of Korea; ^3^Clinic for Pediatric Cardiology, Saarland University Hospital, 66421 Homburg, Germany; ^4^Inorganic Chemistry Department, Saarland University Campus C4 1, 66123 Saarbrücken, Germany; ^5^Korea Institute of Science and Technology Europe (KIST-Europe) Forschungsgesellschaft mbH, Campus E 7 1, 66123 Saarbrücken, Germany

## Abstract

A novel synthesis of a nanostructured cell adhesive surface is investigated for future stent developments. One-dimensional (1D) Al_2_O_3_ nanostructures were prepared by chemical vapor deposition of a single source precursor. Afterwards, recombinant filamentous bacteriophages which display a short binding motif with a cell adhesive peptide (RGD) on p3 and p8 proteins were immobilized on these 1D Al_2_O_3_ nanostructures by a simple dip-coating process to study the cellular response of human endothelial EA hy.926. While the cell density decreased on as-deposited 1D Al_2_O_3_ nanostructures, we observed enhanced cell proliferation and cell-cell interaction on recombinant phage overcoated 1D Al_2_O_3_ nanostructures. The recombinant phage overcoating also supports an isotropic cell spreading rather than elongated cell morphology as we observed on as-deposited Al_2_O_3_ 1D nanostructures.

## 1. Introduction

Stent angioplasty is accepted as one of the breakthrough inventions for the treatment of arterial occlusion which usually leads to a fatal complication [1]. On the other hand it is still a challenge to enhance the endothelialization on the surface of the stent in order to prohibit any restenosis after the implantation [2]. Therefore, the development of biocompatible surfaces which promotes the adhesion and the proliferation of endothelial cells is of great interest. In this context various surface modification methods have been investigated to control the cellular response on stent materials without altering their bulk properties [3, 4]. Among other surface properties, the topography plays a major role in the endothelialization as we showed previously [5]. Nanotopography enhances the adhesion and the proliferation of endothelial cells on the substratum.

In addition to the surface topography, chemical surface modification by biopassive and/or -active coatings promotes the cellular response [6]. Such a biochemical modification especially may help hindering the early immune responses including inflammation and the encapsulation [6, 7]. For instance, the modification of the surface by fibrin and laminin helps in reduction of any possible foreign body responses. Fibrin and laminin are extracellular matrix proteins (ECMs) and it is known that they are involved in early immune response processes such as inflammation. Various experimental studies show that a coating of medical implants with these proteins leads to improvement in the tissue integration and regeneration [8, 9].

Most recently, genetically engineered filamentous bacteriophage has been introduced as an effective tool for biochemical surface modification especially in targeted drug and antibody delivery as well as tissue engineering applications [10, 11]. Such bacteriophages may act as linkers for the attachment of biomolecules on the implant materials to control the cellular responses. The fd-phage is a member of Ff filamentous phage family consisting of a circular single-stranded DNA (ssDNA) and a surrounding capsid protein. The capsid protein is mainly composed of the major coat protein, p8 (~2700–3000 copies), aligned along the ssDNA. Five copies of minor coat protein, p3, are located at one end of the fd filament and five copies of p7 and p9 proteins are expressed at the other end, the recombinant phage (re-phage) is an fd-tet derived phage of which a gene cassette encoding recombinant p3 and p8 proteins is inserted into the fd-tet plasmid [11].

In this study, we investigate the effect of the re-phage on the cellular response in addition to the surface topography of 1D Al_2_O_3_ nanostructures. Cell adhesive molecules (RGD peptides) and transition metal oxides binding molecules (CHRRPSRSC peptide) are genetically engineered on the P8 and P3 capsid proteins of re-phage for the improvement of cell binding and phage immobilization on 1D Al_2_O_3_ nanostructures, respectively. We compared the early response of endothelial cells on as-deposited and re-phage overcoated 1D Al_2_O_3_ nanostructures for future stent applications.

## 2. Materials and Methods

### 2.1. Genetic Modification and Amplification of Phage

The N-terminus of recombinant p8 and p3 of fd-tet plasmid (kindly provided by Professor Dr. George P. Smith, University of Missouri, USA) was used to introduce two genes, encoding RGD peptide for cell adhesion and CHRRPSRSC peptide for binding with 1D Al_2_O_3_ nanostructures [12]. The sequences of adaptor DNA fragments were constructed using the complementary oligonucleotides as follows:

1. RGD oligonucleotides:
   forward primer: 5′-AGCTTT TGT AGG GGT GAC GGT AGG TGC GGTAC-3′;
   reverse primer: 5′-C GCA CCA ACC GTC ACC CCT ACA AA-3′;
2. 1D Al_2_O_3_ nanostructures binding oligonucleotides:
   forward primer: 5′-AGCTTTCATCGCCGCCCGAGCCGCAGCGGTAC-3′;
   reverse primer: 5′-CGCTGCGGCTCGGGCGGCGATGAA-3′.

1D Al_2_O_3_ nanostructures binding peptide (CHRRPSRSC) was introduced in the p3 of plasmid. Subsequently, cell adhesive peptide (RGD) was introduced for the recombinant p8 of plasmid. The recombinant plasmid was digested by HindIII/KpnI and SfiI/NotI (New England Biolabs, Germany) for N-terminus of recombinant p8 and p3 genes, respectively.

Each of the two complementary oligonucleotide mixtures was boiled for 10 min and cooled down to room temperature to produce an adaptor DNA. Isolation of wild-type phages (wt-phage) and re-phage from the host cells was performed by the polyethylene glycol/sodium chloride (PEG/NaCl) precipitation method, as described elsewhere [13]. After growing the transformed K91BK cells in Luria Broth supplemented with tetracycline (20 *μ*g/mL) and kanamycin (100 *μ*g/mL) at 37°C with vigorous shaking (260 rpm) overnight, final phage pellets were dialyzed with 30 kDa cut-off membrane against 10 mM phosphate buffered saline solution (PBS) at pH 7.4 overnight to remove the remaining PEG. The phage concentration (colony-forming units per milliliter, cfu/mL) was determined by the phage titration. Phage suspensions were stored at 4°C. The concentration was diluted with distilled-deionized water (ddH_2_O) to a phage stock concentration of 1 × 10^10^ cfu/mL. A photospectrometer (Evolution 60, UV/Vis) was used to estimate the concentration precisely.

### 2.2. Synthesis and Coating of 1D Al_2_O_3_ Nanostructures with Recombinant Phage

**Figure 1:**
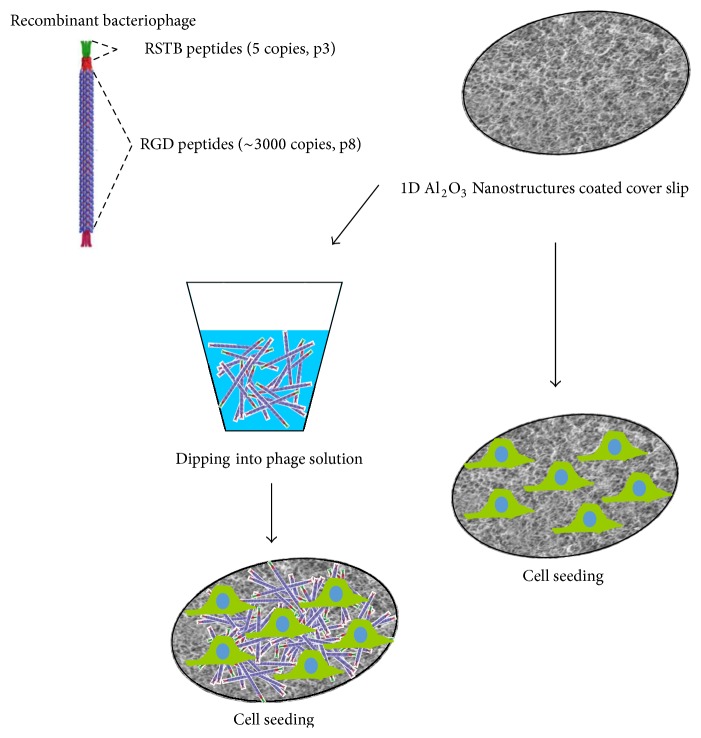
Schematic illustration of the fabrication method for bioactive coating on 1D Al_2_O_3_ nanostructures.

1D Al_2_O_3_ nanostructures were deposited on round shaped glass cover slips (*d* = 12 mm, Carl Roth, Germany) by the decomposition of a single source precursor [^t^BuOAlH_2_]_2_ at 600–650°C in a cold-wall CVD chamber under vacuum conditions. The complete deposition process and the synthesis of the precursor were described in detail previously [14, 15]. Afterwards, we applied recombinant to coated glass using a simple dip coating process. Previously, Choi et al. identified a TiO_2_-specific STB1 peptide (CHKKPSKSC) using the phage display technique and RSTB (CHRRPSRSC), which was specialized to control the growth of TiO_2_ [12]. Following the similar approach (metal-oxide-specific binding), 1D Al_2_O_3_ nanostructures are incubated at 37°C for 30 min in phage solution of concentration at 1 × 10^10^ cfu/mL. Prior to seeding the cells, each sample was washed using PBS with 0.05% Tween 20 for removal of nonspecific binding of phage as shown in Figure 1.

### 2.3. Cell Culture and Microscopic Analysis

Human endothelial EA hy.926 cells (ATCC: CRL-2922, USA) were seeded on as-deposited and on re-phage coated 1D Al_2_O_3_ nanostructures following the protocol as described elsewhere [16]. Bare glass cover slips were used as control. Following an incubation time of 24 h at 37°C, cell and phage mixtures were observed by immunofluorescent staining. Biotin conjugated anti-fd phage antibodies and F-actin (1 : 5000, Sigma) were added and incubated for 1 h at 37°C. Vinculin (Invitrogen, Germany) and 4′,6-diamidino-2-phenylindole (DAPI, Amersham, Germany) were subsequently stained with the cells. After the antibody incubation, the samples were mixed with Cyanine dye-3- (Cy3-) conjugated streptavidin solution (1 : 1000, Sigma) and Cyanine dye-5- (Cy5-) conjugated secondary antibody solution (1 : 1000, Sigma) with staining were visualized with the Olympus IX51 inverted microscope with excitation wavelength at 550 and 660 nm, respectively. To evaluate the cell viability with the re-phages, WST-1 assay (Roche Applied Science, Switzerland) was performed according to the instructions provided by the manufacturer [17]. The results of cell viability are represented as the mean and standard deviation (±SD) from three independent experiments, with significance of differences evaluated using ANOVA with Fisher's post hoc comparisons. A probability of *P* = 0.05 was considered significant for all tests.

### 2.4. Scanning Electron Microscope (SEM) Imaging

As-deposited and re-phage over coated 1D Al_2_O_3_ nanostructures were sputtered with gold-palladium (Polaron, Sputter Coater) and analyzed under SEM (Quanta FEI-SEM, Holland). For the cells, 24 h cultured cells on prepared substrates were fixed following our standard protocol: substrates were washed 2 times with PBS at 37°C and the fixation was done using 2.5% glutaraldehyde in 0.5 M cacodylate buffer with 6% sucrose for 2 h at room temperature under movement. Afterwards, the substrates were incubated in osmium tetroxide (4% in deionized water (dH_2_O)) for 2 h under movement in a dark chamber. The substrates were stored in dH_2_O at 4°C overnight. The water was removed by washing substrates twice in ethanol under movement at 4°C for 5 min (30%, 50%, 70%, 80%, and 90%). Dehydrating was finished by washing 3 times in ethanol (100%) for 15 min under movement at 4°C. The substrates were subjected to critical point drying (Polaron CPD 7501, Quorum Technologies, Ringmer, UK). Afterwards, they were sputtered with gold-palladium and visualized under SEM.

## 3. Results and Discussion

### 3.1. Immobilization of Re-Bacteriophage on 1D Al_2_O_3_ Nanostructures

**Figure 2:**
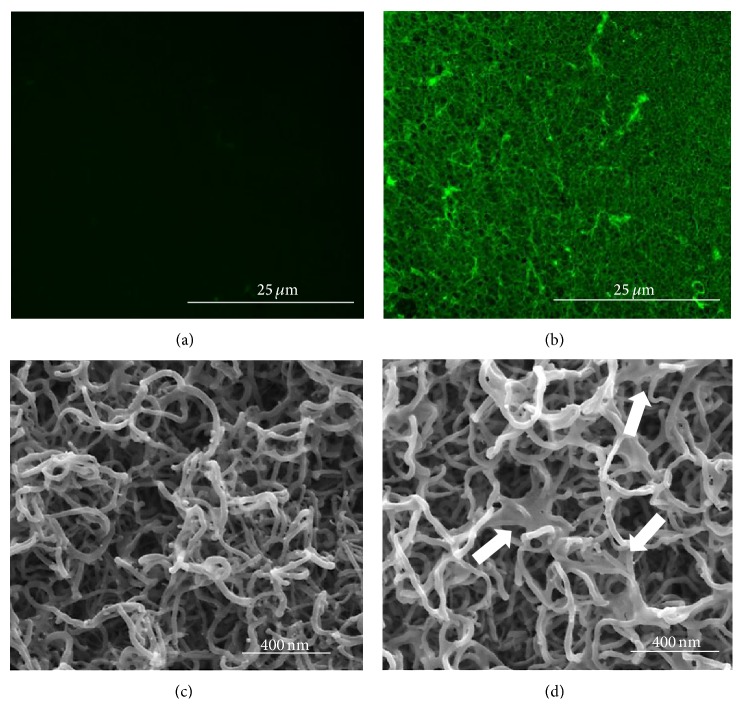
Fluorescence and SEM images of wt-phage ((a) and (c)) and re-phage coated 1D Al_2_O_3_ nanostructures ((b) and (d)), respectively. Phages were stained with anti-M13 (green) antibody. Arrows in (d) indicate film-like re-phage assemblies.

1D Al_2_O_3_ nanostructures exhibit a chaotic nature and reveal a highly porous nature as shown in Figure 1 and more detailed in Figure 2. Previously, we have shown that such nanostructures are composed of an inner Al core covered by Al_2_O_3_ shell [15]. As shown in Figure 2, fluorescence microscope and SEM analyses show the wt-phage and re-phage coated 1D Al_2_O_3_ nanostructures. It is clearly seen that wt-phages were not bound to the deposited nanostructures and they were flushed away after the washing step as shown in Figures 2(a) and 2(c). In contrast, the re-phages were successfully immobilized on 1D Al_2_O_3_ nanostructures (Figures 2(b) and 2(d)). Selective binding of re-phages to the 1D Al_2_O_3_ nanostructures results in high fluorescence intensity contrast (cross-linking structure) and SEM images of re-phage on 1D Al_2_O_3_ nanostructures revealed self-assembling process. We observed that the re-phages layer was formed as thin film-like coverage over 1D Al_2_O_3_ nanostructures as indicated by arrows in Figure 2(d). Previously, Choi et al. identified a TiO_2_-specific polar peptide (CHRRPSRSC) using phage display technique [12]. Similarly, we believe that the binding of peptide, giving rise to a theoretical isoelectric point of 10.41 to 1D Al_2_O_3_ nanostructures, may arise from electrostatic interaction between positively charged three arginine and one histidine residues and negatively charged oxides surface.

### 3.2. Cell Adhesion and Morphology Changes on Prepared Surfaces

**Figure 3:**
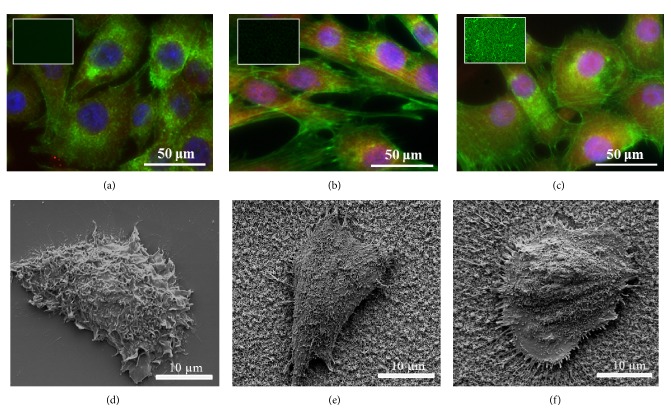
Fluorescence and SEM images of human endothelial EA hy.926 cells stained with F-actin (green), vinculin (red), and DAPI (blue) on glass ((a) and (d)), 1D Al_2_O_3_ nanostructures ((b) and (e)), and re-phage coated 1D Al_2_O_3_ nanostructures ((c) and (f)) after 30 h, respectively. Inset indicates anti-M13 green fluorescence.

The cell morphological changes were investigated by the observation of the focal adhesion on the bare and re-phage overcoated 1D Al_2_O_3_ nanostructures as shown in Figure 3. Cell adhesion markers, F-actin and vinculin, indicate that cells interact strongly with 1D Al_2_O_3_ nanostructures. In general, enhanced cell adhesion is associated with cell spreading and actin fiber on surface of materials. While no actin fibers were observed on cell seeded bare glass substrate after 30 h cell culture, actin fibers are clearly seen on both as-deposited and re-phage overcoated 1D Al_2_O_3_ nanostructures. Cells seeded on Al_2_O_3_ 1D nanostructures exhibit an elongated morphology which may occur due to the interaction with the surface topography. On the other hand we observed that cells seeded on re-phage coated Al_2_O_3_ 1D nanostructures spread over the surface (without any elongation in a preferential direction) and they exhibit a round shaped morphology. We do believe that such a difference in the morphology can be attributed to the synergetic effect of the re-phage overcoating and the nanotopography.

### 3.3. Cell Proliferation Analysis

**Figure 4:**
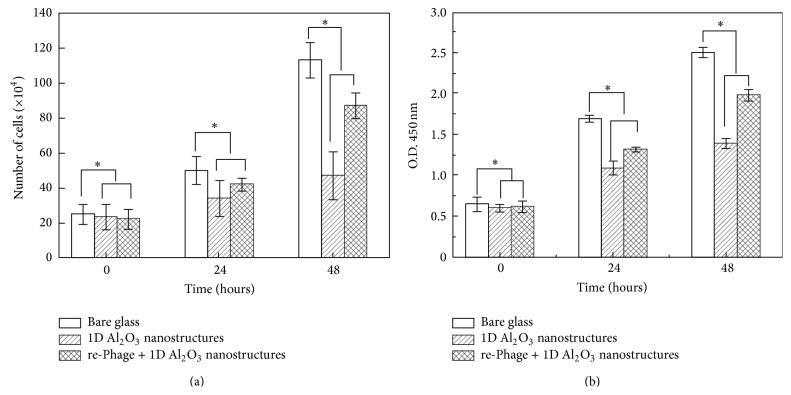
Cell proliferation rate as determined by cell counts (a) and WST-1 assay (b). Cell number was determined using ImageJ. Data were analyzed statistically by ANOVA at a *P* < 0.05 confidence interval and Fisher's LSD test. Asterisk denotes differences of the populations (^∗^ *P* < 0.05 compared to respective controls).

The cell proliferation and viability results (three times from 0 to 48 h) are shown in Figure 4. The initial cell culture at the beginning (0 h) showed similar number of cells and cell viabilities. However, cell cultured on 1D Al_2_O_3_ nanostructures from 24 to 48 h exhibited the lowest cell proliferation and population. We observed a decrease in cell viabilities and populations on 1D Al_2_O_3_ nanostructures in comparison to cells cultured on bare glass as control.

The decrease in cell viabilities and populations can be explained by the difference in the surface topography induced by the deposited 1D Al_2_O_3_ nanostructures [18, 19]. However, overcoating of 1D Al_2_O_3_ nanostructures with re-phages led to a clear increase in cell populations and viabilities around 40% and 30%, respectively, as compared to 1D Al_2_O_3_ nanostructures.

## 4. Conclusions

This preliminary study showed that re-phage coated 1D Al_2_O_3_ nanostructures provide an enhanced endothelial cell adhesion and proliferation which can be interesting for future stent applications in cardiology. In our study, we successfully demonstrated incorporating chemically/genetically engineered phage assemblies on 1D Al_2_O_3_ nanostructures. Our approach using recombinant phages may lead to further functionalization of the surface by other biomolecules. This may add synergetic effect to the topography controlled cellular response.
